# An Analysis of the Use of Proparacaine in Cataract Surgery

**DOI:** 10.7759/cureus.22175

**Published:** 2022-02-13

**Authors:** Allen Dang, Akshay J Reddy, Vivek Pokala, Joel Rabara, Hetal Brahmbhatt

**Affiliations:** 1 Anaesthesia, California Northstate University, College of Medicine, Elk Grove, USA; 2 Opthalmology, California Northstate University, College of Medicine, Elk Grove, USA; 3 Health Sciences, California Northstate University, Rancho Cordova, USA; 4 Medicine, California Northstate University, College of Medicine, Elk Grove, USA; 5 Psychiatry, Mercy General Hospital, Sacramento, USA

**Keywords:** injection, lidocaine, surgery, cataracts, proparacaine

## Abstract

A cataract is the primary cause of preventable blindness and is characterized by a congenital, developmental, or acquired opacity of the human lens. Cataracts are predominantly treated through surgical procedures utilizing a combination of anesthetic agents such as proparacaine to reduce patient discomfort. Proparacaine is used to inhibit voltage-gated sodium channels on neuronal membranes to prevent signal propagation and pain signaling in the patient. Current clinical standards call for the utilization of 0.5% proparacaine when used for local anesthesia in cataract surgeries. In this review, the authors extracted the reported application site and concentrations of proparacaine in conjunction with various combination agents to accurately describe its usage in cataract surgery. It was found that most surgeons adhered to the standard concentrations of proparacaine and generally used tropicamide, an eye dilator, as a combination agent in cataract surgery. Additionally, surgeons preferred anesthetic application to the retrobulbar block. The authors find that although surgeons are following standard protocol, adjustments for lowering the standard dose of proparacaine could prove beneficial in preventing proparacaine toxicity. Furthermore, the authors find that more research can be conducted in the future examining other combination agents for use with proparacaine to improve patient outcomes.

## Introduction and background

When the lens of a patient's eye becomes hazy, it is referred to as a cataract. Cataracts are most commonly caused by the aging process. Although cataracts are treatable, numerous individuals across the globe suffer from this condition. In fact, one out of every two people above the age of 74 is thought to have a cataract [1]. The collagen within the eye's lens deteriorates with time as a result of ultraviolet (UV) radiation exposure, causing the normally clear lens to become cloudy in appearance. For instance, one study found that excessive UV exposure significantly increases the risk of cataract development [2]. This degradation caused by UV exposure might result in cloudy vision and decreased nighttime perception. A patient's cataracts can be surgically removed and replaced with an intraocular lens (IOL). Globally, millions of cataract procedures are projected to be performed each year [3]. Anesthetics are frequently used by surgeons to reduce the amount of discomfort that patients may suffer during an operation. Traditionally in cataract surgery doctors will use medications such as tetracaine, lidocaine, bupivacaine, and proparacaine to numb a patient's eye before the surgery begins [3,4]. Ophthalmologists also utilize tropicamide to dilate a patient's eye before surgery [3]. Proparacaine, which is an active anesthetic agent, is one of the most commonly used anesthetics for this surgery [4]. Because this anesthetic is often used for this operation, it is critical to outline and understand the correct standards and protocols that must be followed when using it to protect and maintain patient health. In order to achieve this objective, this paper attempts to analyze the application, dosage, and concentration of proparacaine, as well as the types of other combination agents that are used during the cataract surgery procedure. The goal of this study is to look at how proparacaine is currently used and applied to patients undergoing cataract surgery.

## Review

Concentration 

The concentration of proparacaine needed to be administered during cataract surgery can depend upon a plethora of factors, including dosage (number of drops), patient physiology, and clinical regulations. Current research describing the use of proparacaine in standard cataract surgeries describes a concentration of 0.5% as the golden standard [5,6]. Incidentally, the most common proparacaine concentration used within the review was at 0.5%. According to the data collected from Table 1, 24 of the studies within the review states the use of concentrations of 0.5% [7-32]. This concentration would likely indicate the bare minimum amount of anesthetic to achieve a significant effect on a standardized patient. As physicians, it is imperative to be updated with current standards of care and their application to direct patients. This is needed to best ensure the safety of the patient and their clinical outcomes. With that being said, it is unsurprising to see most of the studies within this review use proparacaine concentrations of 0.5% during cataract surgery as it follows the standard of care for the standardized patient that was established by physician consensus and legal documentation. Although most of the studies would point to the use of 0.5% concentrations of proparacaine, a minority of studies indicate a preference for higher and lower proparacaine concentrations of 1% and 0.01%, respectively [17-18,29]. This most likely points towards the notion that patients hold differing thresholds to achieve a significant analgesic effect or that distinct procedures with unique combination agents need varying concentrations of proparacaine to reinforce patient wellbeing. Although the articles did not highlight specific reasons, a higher concentration at 1% was required to ensure the patient was placed into an analgesic state during surgery [17,29]. A lower concentration at 0.01% could have been implemented with the patient's safety in mind [18]. Side effects of the ester proparacaine are generally limited; however, proparacaine toxicity has been widely mentioned in the literature. Proparacaine toxicity mainly stems from chronic use of the anesthetic; however, the development of toxic outcomes on the corneal epithelium has been mentioned even after single applications of proparacaine. According to a recent study, using lower concentrations of local anesthetic compared to the standard of care is seen to have lower risks of developing proparacaine toxicity [6]. In medicine, the standards of care for surgical operations are constantly changing, and as physicians, it is vital to understand when modifications of treatment are required to best suit the needs of the patient. 

**Table 1:** The use and application of proparacaine during cataract surgery

Author (year)	Concentration	Combination agents	Area(s) of application	Dosage
Duffin et al. (1982) [7]	0.50%	Tetracaine	Retrobulbar	6 drops
Erdurmus et al. (2008) [8]	0.50%	N/A	Conjunctival sac	3-4 drops
Erza et al. (1996) [9]	0.50%	Lidocaine, bupivacaine	Retrobulbar	2-4 drops
Habib et al. (2004) [10]	0.50%	N/A	Conjunctival sac	N/A
Hamilton et al. (1998) [11]	0.50%	Tetracaine	Retrobulbar	1 drop
Ho et al. (1992) [12]	0.50%	Tropicamide	Retrobulbar	N/A
Ioannidis et al. (2010) [13]	0.50%	Lidocaine, lignocaine	Retrobulbar	2 drops
Joshi (2014) [14]	0.50%	Lidocaine	Retrobulbar	1 drop
Judge et al. (1997) [15]	0.5%	Bupivacaine, tetracaine, lidocaine	Retrobulbar	N/A
Kim et al. (2015) [16]	0.50%	Tromethamine	Retrobulbar	3 drops
Khokhar et al. (1997) [17]	1%	Tropicamide, phenylephrine	Retrobulbar	3 drops
Caporossi et al. (2014) [18]	0.01%	N/A	Transepithelial corneal injection	N/A
Ong-Tone (2003) [19]	0.50%	Cyclopentolate, phenylephrine, ofloxacin, flurbiprofen	Retrobulbar	N/A
Mannan et al. (2017) [20]	0.50%	Lignocaine	Retrobulbar	3 or 4 drops
Matthew et al. (2002) [21]	0.50%	N/A	Retrobulbar	3 or 4 drops
Matthew et al. (2003) [22]	0.50%	Lignocaine, bupivacaine	Retrobulbar sub-Tenon	N/A
McCormick et al. (2006) [23]	0.50%	Tropicamide 1%, phenylephrine 2.5%, atropine 1%	Conjunctival sac	1 drop
Meyer et al. (1992) [24]	0.50%	Tropicamide 1%, phenylephrine hydrochloride (HCl) 2.5%	Retrobulbar	N/A
O’Brien et al. (2005) [25]	0.50%	Tropicamide 1%, phenylephrine hydrochloride 2.5%, cyclopentolate 1%	Retrobulbar	2 drops given three times in two minutes intervals
Oguz et al. (2000) [26]	0.50%	N/A	Retrobulbar	1 drop
Ruschen et al. (2005) [27]	0.50%	Tetracaine 1%	Retrobulbar	2 drops
Spanggord et al. (2005) [28]	0.50%	N/A	Retrobulbar	2 drops
Sugar (1998) [29]	1%	N/A	Retrobulbar	N/A
Westermeyer et al. (2018) [30]	0.50%	Subconjunctival lidocaine 2%+	Retrobulbar	2 drops every two minutes up to five times
Wollensak et al. (2009) [31]	0.50%	N/A	Retrobulbar	2 drops every five minutes for 30 minutes
Zaheer et al. (2007) [32]	0.50%	Intracameral lignocaine 1%, sub-Tenon's anesthesia	Retrobulbar	4 drops

Combination agents

**Figure 1:**
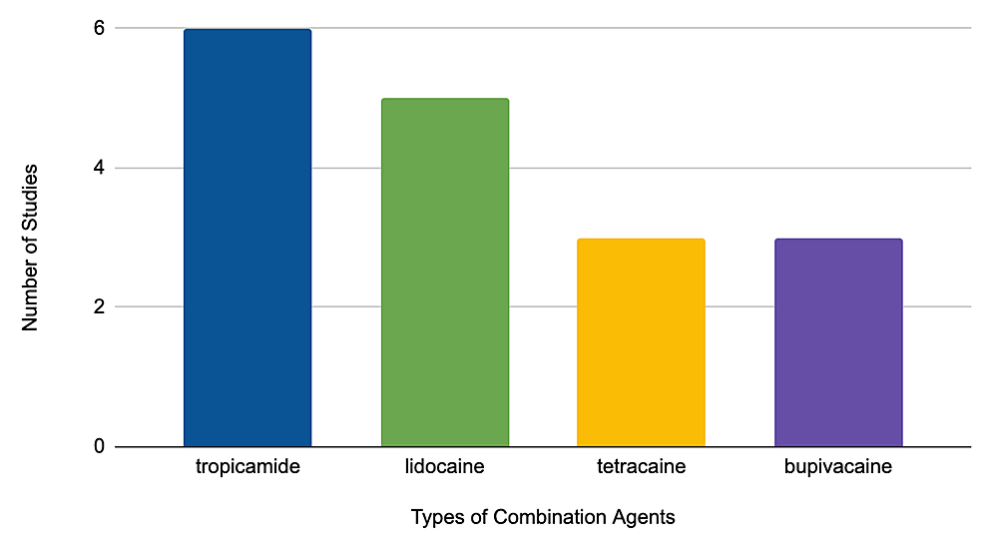
Frequency of combination agents in different studies

Although proparacaine can be used as the principal anesthetic agent, it is generally used with other combination agents to ensure that patients do not feel any pain while they are going through cataract surgery [7]. The review found that six of the studies reported using tropicamide in conjunction with proparacaine [12,17,23-25]. This was most likely done because tropicamide is used in ophthalmology as a pupil dilator. Tropicamide is not an anesthetic used to numb a patient's eye during surgery; rather, it is a chemical used to dilate the pupil to ensure that the surgical procedure is done in a proper manner [7]. Ophthalmologists need to be able to have a clear view of the tissues behind the iris when performing cataract surgery; this can only be done if the patient's pupil is relatively large. A large number of investigations within the review most likely report the usage of tropicamide because patients' pupils before surgery were not probably dilated enough to provide the surgeon with a decent view of the structures behind the iris of the patient. Another major combination agent that was reported to be used in conjunction with proparacaine during cataract surgery was lidocaine. According to the data presented in Figure 1, five of the research articles reported using lidocaine in combination with proparacaine [9,13-15,30]. Lidocaine is an amino amide local anesthetic that can be used for various different surgical procedures. Physicians most likely used this anesthetic in conjunction with lidocaine, which creates a mixture of an amino ester and an amino amide, to produce a stronger analgesic effect on the patient so that they would not experience significant harm during the procedure [9]. Another anesthetic that was found to be used in concurrence with proparacaine was tetracaine. Three studies within the review found that tetracaine was being used as a major combination agent during cataract surgery [7,11,27]. Although both lidocaine and tetracaine are powerful anesthetic agents, lidocaine is used more than tetracaine during cataract surgery because tetracaine takes a longer period of time to produce an analgesic effect than lidocaine [6]. For the purposes of cataract surgery, it might be more practical to produce an immediate, 30 seconds to onset, an analgesic effect so that the procedure can start immediately. In addition to tetracaine, bupivacaine was also reported to be used in combination with proparacaine to produce an analgesic effect. Bupivacaine was discovered to be a major combination agent during cataract surgery in three studies included in the review [9,15,22]. Like tetracaine, bupivacaine takes a longer time to produce an analgesic effect than lidocaine [9]. As a result, while bupivacaine is used as a combination agent in a great number of cataract surgery procedures, lidocaine is still used more frequently due to its fast-acting nature as an analgesic.

Application of anesthesia

For ophthalmologists who perform cataract surgery, it is critical not only that they utilize the correct type of anesthesia but also that they ensure the application of anesthetic to the correct location. Even if surgeons use the right type of anesthesia but don't administer it correctly, the patient would experience excruciating pain during surgery [4]. As a result, understanding how anesthesia is used is critical when trying to enhance patient care for people who have cataract surgery. According to the data presented in Table 1, 23 research papers reported that retrobulbar injections were the most common method for applying proparacaine [7-32]. This is likely because, in order for physicians to successfully perform the procedure and minimize harm to the patient, they need relaxation of the muscles around the eye to ensure as little movement as possible during the procedure. Proparacaine is able to achieve this state of muscle relaxation by temporarily blocking the functionality of cranial nerves III, IV, and VI, which control ocular movement. Although this is the primary proparacaine application method during cataract surgery, some risks come with retrobulbar injections. Retrobulbar blocks cause complications, including reduced visual acuity, vitreous hemorrhaging, retinal detachment, cardiac arrest, seizures, and even death [11,16]. Therefore it is important that physicians follow proper procedural protocol when administering retrobulbar injections to minimize patient harm and maximize patient health. While the potential complications that could happen from retrobulbar injections may result in morbidity and mortality, they rarely occur due to the current standards and clinical practices implemented by ophthalmologists [11]. If the risk to benefit ratio of retrobulbar blocks is unreasonably high, physicians can utilize other methods of administration for proparacaine in cataract surgeries. A recent peer-reviewed article has stated that topical anesthetics can be used safely for cataract extraction [5]. It was found that there was no statistical difference in surgical conditions between groups given with either topical or retrobulbar anesthesia. However, there was marginally higher discomfort during the administration of proparacaine and postoperatively. Several other locations of proparacaine administration were present within the data seen in Table 1. Specifically, three articles were found to use topical administration on the conjunctival sac [8,10,23]. Besides avoiding possible side effects of retrobulbar injections, potential advantages in using topical administration of proparacaine are well established and include a more rapid return of patient ambulation, quicker surgeries, and the ability to conduct an outpatient surgery [6]. However, topical proparacaine can rarely cause side effects such as a tonic-clonic seizure that could potentially cause long-term side effects. Although most of the drawbacks of both the retrobulbar and topical anesthesia techniques are rare, it is important to become aware of them in determining which administration type fits the patient [16,18]. Anesthesia is vastly important to the outcome of cataract surgery, and thus it is exceptionally important to pick the right procedure for the patient.

Current limitations and future applications

Despite all of the existing research on the administration and use of proparacaine in cataract surgery patients, further research is needed to discover more appropriate standards that may be employed for these procedures to avoid adverse effects. More specifically, supplemental research on the effects of proparacaine when used in conjunction with combination agents is necessary. When conducting this research, chemicals such as ethylenediaminetetraacetic acid (EDTA) may be required to see what effects different compounds have on the analgesic effects of proparacaine. Appropriate anesthetic use is critical for reducing the pain that patients may suffer as a result of cataract surgery. On the other hand, individuals with cataracts must be diagnosed early to avoid negative health consequences and diminishing surgical returns. Artificial intelligence (AI) software that analyzes retinal scans could be developed in the future to help enhance the frequency with which cataract patients are detected. Further research into the effects of proparacaine is needed to ensure the safety of cataract surgery patients. Perhaps in the future, investigations could invest in potentially finding a method to prevent cataracts from developing. There could potentially be a lifestyle change that might mitigate the development and progression of cataracts like we have seen for other diseases such as Alzheimer's. Whatever the situation may be, more research into cataracts and cataract surgery is needed to improve patient outcomes.

## Conclusions

Cataract surgery is a critical medical operation that protects many people's vision. Understanding how anesthetics like proparacaine are used during this treatment can assist in enhancing the existing standard of care for patients with this condition. Proparacaine is an anesthetic that functions to relax eye muscles during the procedure so that they generate as little movement as possible. Additionally, this anesthetic also serves to block pain that could be felt during the operation by blocking the functionality of specific nerve endings. After reviewing the literature, lidocaine, tetracaine, and tropicamide were determined to be the most regularly utilized combination drugs with proparacaine in cataract procedures. Although proper protocol for administration of proparacaine during cataract surgery was present within most of the studies, the standard dosage for proparacaine should be reduced to reduce patient harm and improve treatment outcomes. It is hoped that this review will encourage further research into the application of the proparacaine anesthetic during cataract surgery. 
